# Morphological awareness shows a more robust unique association with reading comprehension than phonological awareness in Chinese children in Grades 3–5

**DOI:** 10.3389/fpsyg.2026.1946945

**Published:** 2026-09-21

**Authors:** Weibin Hu, Xueru Zhang, Ling Ding

**Affiliations:** 1 School of Education, Lanzhou City University, Lanzhou, China; 2 School of Rehabilitation and Physical Education, Nanjing Normal University of Special Education, Nanjing, China

**Keywords:** character dictation, Chinese children, lexical representations, morphological awareness, phonological awareness, reading comprehension

## Abstract

Reading comprehension in morphosyllabic Chinese draws on coordinated phonological, orthographic, morphological, and broader language knowledge, but the unique associations of morphological and phonological awareness remain uncertain once proximal reading and language skills are considered. This cross-sectional study examined 361 Mandarin-speaking children in Grades 3–5. Hierarchical regressions predicted sample-standardized reading-comprehension scores after adjustment for grade, sex, school, non-verbal reasoning, decoding, listening comprehension, vocabulary, character dictation, and syntactic awareness. Inference used HC3 robust standard errors, case-bootstrap confidence intervals based on 5,000 resamples, and sensitivity analyses. After all covariates were entered, morphological and phonological awareness jointly accounted for an additional 2.9% of variance, ΔF(2, 346) = 17.65, *p* < 0.001. Morphological awareness showed a unique association with comprehension (β = 0.215, *p* < 0.001), whereas the phonological-awareness coefficient was smaller and non-significant (β = 0.057, *p* = 0.192). The direct coefficient contrast favored morphological awareness (βMA−βPA = 0.158, HC3 *p* = 0.009; bootstrap 95% CI [0.043, 0.275]). Morphological awareness remained significant across all sensitivity specifications. After influential-case exclusion, phonological awareness also became significant, and the coefficient difference remained significant. The findings identify a larger and more stable unique morphological association with reading comprehension in Chinese children in Grades 3–5, while the smaller phonological association was substantially shared with character-level literacy.

## 1 Introduction

Reading comprehension requires readers to identify written words and construct a coherent representation of their meaning across sentences and passages. The Simple View of Reading (SVR) describes comprehension as depending fundamentally on decoding and linguistic comprehension (Hoover and Gough, 1990). These components provide a framework for locating the contribution of metalinguistic skills: awareness of linguistic structure can support word identification, the construction of meaning from language, and the coordination of these processes during reading. In Chinese, a large meta-analytic structural equation model found that metalinguistic skills contributed to decoding and were strongly related to language comprehension, with no additional direct contribution to reading comprehension beyond the two SVR components (Peng et al., 2021). This evidence places metalinguistic skills within the foundations of reading comprehension and raises the question of how particular skills contribute within that broader organization.

The Direct and Indirect Effects Model of Reading (DIER) specifies hierarchical relations among component skills and their direct and indirect connections to comprehension (Kim, 2020). It also proposes that these relations vary with development and assessment demands. This framework helps explain why a skill can support comprehension through word reading and language comprehension while retaining an additional association when the measures differ in their linguistic demands. In the present study, SVR organizes the core reading and language components, DIER informs the proposed relations among skills, and the Lexical Constituency Model specifies the coordination of orthographic, phonological, and semantic information in Chinese character identification (Perfetti et al., 2005).

Chinese is commonly characterized as a morphosyllabic writing system. A character typically represents a syllable and a morpheme, while the mapping from orthography to pronunciation is less transparent than the grapheme–phoneme correspondences found in many alphabetic languages. Modern Chinese also contains many homophonous morphemes and relies heavily on lexical compounding. Consequently, successful reading requires more than access to a phonological form. Readers must distinguish among competing meanings, identify morphemes, and combine them according to productive word-formation patterns. These properties provide a principled reason to expect morphological awareness to be particularly important for Chinese literacy (Ke, 2025; Liu and McBride-Chang, 2010; Perfetti et al., 2005).

Morphological awareness refers to the ability to reflect on and manipulate morphemes and morphological structures. It is a multidimensional construct in Chinese: studies have distinguished lexical compounding, homophone or homograph awareness, semantic-radical awareness, and sensitivity to the structural relations among morphemes (Han et al., 2022; Liu et al., 2013). Homophone and homograph tasks emphasize the differentiation of morpheme meanings when pronunciations or written forms overlap, while radical tasks operate within characters and word-structure tasks emphasize grammatical relations among constituent morphemes. Compounding awareness overlaps with these facets through access to morpheme meanings, yet specifically requires children to select and combine meaningful units into a semantically and structurally appropriate lexical item. Evidence that compounding and homophone awareness are related but show distinct associations with word reading and vocabulary supports this distinction (Liu et al., 2013). Productive compounding was selected in the present study because it directly samples flexible morpheme combination, a process that can support lexical segmentation, inference of unfamiliar compound meanings, and semantic disambiguation during text processing (Kim et al., 2020; Ku and Anderson, 2003). Experimental and longitudinal evidence has linked Chinese morphological awareness to word reading, vocabulary, and reading comprehension (Cheng et al., 2016; Liu and McBride-Chang, 2010; Shu et al., 2006; Wang et al., 2023; Xie et al., 2019).

Productive compounding connects morphological awareness to both SVR components. Recognizing constituent morphemes and their structural relations can support the identification of multi-character words. Using those constituents to learn and interpret compound meanings connects morphological awareness to vocabulary and linguistic comprehension. During text reading, the same operations can support lexical inference when a compound is unfamiliar and help select a contextually appropriate meaning. Kim et al. (2020) found direct and indirect relations between compounding awareness and reading comprehension in Chinese second graders, with indirect pathways through vocabulary, word reading, and listening comprehension. Together with the broader findings of Peng et al. (2021), this evidence motivates examining whether productive compounding provides additional information about comprehension after the measured reading and language foundations have been represented.

Meta-analytic evidence strengthens this account. A synthesis of 46 studies involving 10,793 Chinese students found that morphological awareness had a stronger correlation with reading comprehension than phonological awareness or orthographic skill (Dong et al., 2020). A cross-writing-system meta-analysis found that both morphological and phonological awareness were associated with Chinese reading and that the strength of these associations varied with the reading outcome and task used to operationalize each construct (Ruan et al., 2018). A broader cross-cultural meta-analysis of 204 studies likewise documented a stable relation between Chinese morphological awareness and reading acquisition, while showing that assessment and cultural context contribute to variation in observed associations (Ke, 2025). Recent longitudinal continuous-time evidence further indicates reciprocal and time-sensitive relations between morphological awareness and Chinese reading skills (Wang et al., 2025). These findings make it important to test whether productive compounding remains uniquely associated with comprehension after core reading and language foundations are considered.

Phonological awareness is the ability to identify and manipulate sound units in spoken language, including syllables, onsets, rimes, tones, and phonemes. In Mainland China, Hanyu Pinyin instruction makes onset, rime, and tone information directly relevant to learning and retrieving character pronunciations. These units do not develop identically: syllable-level performance can reach high levels relatively early, whereas onset-rime awareness and Pinyin knowledge continue to develop during the school years (Lin et al., 2020). The present measure therefore sampled rime identification, onset matching, tone identification, and phoneme deletion to represent complementary levels of explicit phonological analysis. In Grades 3–5, these skills can support Pinyin decoding, retrieval of character pronunciations, and the establishment of orthographic-phonological representations. Their unique association with comprehension may nevertheless become smaller as decoding and character-specific literacy become more established. Because identical syllables can correspond to numerous morphemes and characters, phonological information alone frequently underdetermines lexical meaning (Dong et al., 2020; Ho et al., 2004; Ruan et al., 2018; Shu et al., 2006).

Phonological awareness is expected to relate to reading comprehension chiefly through its support for pronunciation analysis, word identification, and access to lexical representations. In Chinese, phonological analysis helps distinguish and retrieve pronunciations that must be coordinated with character forms and meanings. The Lexical Constituency Model locates these operations within interconnected phonological, orthographic, and semantic constituents (Perfetti et al., 2005). This account predicts overlap with decoding and character-level literacy, alongside relations with vocabulary and listening comprehension. It also predicts that the remaining phonological association will depend on which aspects of that literacy foundation are represented in the model. Comparing phonological and morphological awareness therefore addresses how sound analysis and morpheme-based meaning construction are situated within the broader reading system.

Direct comparisons of morphological and phonological awareness require a common statistical frame. In the present models, both observed scores were converted to z scores using the mean and standard deviation of the full study sample, and their associations with the same sample-standardized reading-comprehension outcome were estimated after the same covariates were entered. Their coefficients therefore compare conditional associations per sample standard deviation, not equivalence of task demands, total cognitive importance, or causal pathways. This distinction is important because control variables can overlap asymmetrically with the focal measures. Character dictation requires coordinated retrieval of phonological, orthographic, and semantic information and can share substantial variance with phonological awareness. Evidence that character writing strengthens orthographic learning further supports treating dictation as a proximal character-level literacy measure (Guan et al., 2011). The Lexical Constituency Model describes high-quality character representations as integrating these constituents during identification (Perfetti et al., 2005). Placing character dictation before the focal predictors provides a direct test of whether morphological and phonological awareness retain unique associations beyond this proximal literacy performance.

Syntactic awareness presents a complementary control. Reading comprehension extends beyond word identification to the integration of propositions and discourse relations. Chinese has little inflectional morphology, making word order and function words important signals of sentence structure. Syntactic skills have predicted sentence and passage comprehension among Chinese elementary school children (Chik et al., 2012; Yeung et al., 2013). Including syntactic awareness helps separate the contribution of word-internal morphological knowledge from a broader sensitivity to linguistic structure.

The present study examined the differential unique associations of morphological and phonological awareness with reading comprehension in Chinese children in Grades 3–5. Hierarchical multiple regression addressed whether the two focal skills provided additional information as theoretically related abilities were represented in successive blocks. Reading comprehension was assessed across sentence, paragraph, and text levels. Model 1 controlled grade, sex, school, and non-verbal reasoning. Model 2 added decoding and listening comprehension as measures of the SVR components, together with vocabulary as a foundation of lexical meaning. Model 3 added character dictation to represent integrated character-level literacy performance and syntactic awareness to represent sensitivity to sentence structure. These measures further specify the character and language abilities with which the focal skills may overlap. Model 4 entered morphological and phonological awareness together to estimate their incremental contribution and compare their unique associations under the same covariate set. Syntactic awareness is itself metalinguistic; its earlier entry reflects its role as a sentence-level control in this comparison of the two focal skills.

Three hypotheses were tested. First, morphological and phonological awareness were expected to make a significant joint contribution after demographic variables, non-verbal reasoning, decoding, listening comprehension, vocabulary, character dictation, and syntactic awareness were controlled. Second, morphological awareness was expected to retain a unique positive association in this fully adjusted model. Third, the morphological-awareness coefficient was expected to be larger and more robust across model specifications than the phonological-awareness coefficient. The third hypothesis concerns the relative magnitude and stability of conditional associations in Grades 3–5.

## 2 Materials and methods

### 2.1 Participants

Participants were recruited from four primary schools in two prefecture-level cities in Gansu Province, China. The schools included two urban and two rural schools. The initial sample comprised 363 children in Grades 3–5. Two children with a reported hearing or intellectual disability were excluded, leaving 361 participants. The final sample included 108 third graders, 168 fourth graders, and 85 fifth graders; 186 were boys and 175 were girls. Mean age was 9.68 years (SD = 0.80, range = 8.10–12.50).

The study was approved before data collection by the Scientific Research Ethics Committee of the School of Education, Lanzhou City University (protocol 2025017; 21 April 2025) and was conducted in accordance with the Declaration of Helsinki. Written informed consent was obtained from the parents or legal guardians of all participating children before participation.

### 2.2 Procedure

Testing was conducted at the participating schools. Group-administered paper-and-pencil measures were completed in the children’s classrooms. Measures requiring oral response were administered individually in a school meeting room. Standard Mandarin recordings accompanied the group measures for which auditory presentation was required. Test administrators were senior undergraduate students in psychology or education who received standardized training before each assessment. To reduce fatigue, no testing session exceeded 40 min. The complete battery was distributed across sessions; only the variables relevant to the present research questions are reported below.

### 2.3 Measures

#### 2.3.1 Reading comprehension

Reading comprehension was assessed at text, paragraph, and sentence levels using materials selected or adapted from previously developed Chinese reading measures (Geng, 2014; Long, 2020; Wen, 2005). The text-comprehension task contained three short passages and 26 items assessing local sentence meaning, contextual integration, coherence inference, authorial intention, important information, and construction of an overall text representation (Cronbach’s α = 0.83). A paragraph-comprehension task contained eight items assessing text understanding and semantic inference (α = 0.74). A sentence-comprehension task contained eight items assessing word-meaning retrieval, pronoun interpretation, and semantic inference (α = 0.74). Each correct response received one point. The primary reading-comprehension score was the sum of all 42 items (possible range 0–42), so items rather than subtests were equally weighted. This raw total was converted to a z score using the mean and standard deviation of the full study sample before regression analysis. A principal-component score based on the three subtests was examined in sensitivity analysis to evaluate whether the unequal subtest lengths affected the focal conclusions.

#### 2.3.2 Decoding

Decoding was represented by timed Pinyin-, character-, and word-reading tasks developed with reference to timed-reading paradigms described in prior literacy research (Ho et al., 2017; Kim, 2023; Meng and Li, 2024). Each task contained 160 stimuli arranged in a 10 × 16 matrix and was administered individually for 1 min. The Pinyin task included randomly generated legal syllables, including whole-syllable forms. The character-reading task sampled 160 characters from the Basic Character List in the 2022 Chinese Language Curriculum Standards (Ministry of Education of the People’s Republic of China, 2022). The word-reading task comprised 160 common two-character words selected by frequency from the Modern Chinese Common Word List (Modern Chinese Common Word List Research Group, 2008). The score for each task was the number read correctly in 1 min. Because the three stimulus types differed in difficulty and score dispersion, their raw scores were standardized separately across the full study sample and then averaged, giving each decoding task equal weight. The resulting equal-weight composite was standardized again across that sample before regression so that its coefficient represented a one-standard-deviation difference on the final analytic composite.

#### 2.3.3 Listening comprehension

Listening comprehension was assessed with sentence and dialogue tasks developed using materials from a Chinese reading-and-listening battery and Chinese proficiency tests (Center for Language Education and Cooperation, 2026; Long, 2020). The sentence task contained 10 multiple-choice items presented twice in standard Mandarin (α = 0.79). The dialogue task contained 10 short dialogues, each followed by a picture-selection item and presented twice (α = 0.71). Each correct response received one point. Because the two subtests had the same number of items, score range, and scoring rule, their raw scores were summed (possible range 0–20) and the total was standardized across the full study sample before regression analysis.

#### 2.3.4 Vocabulary knowledge

Vocabulary knowledge was measured with a 40-item breadth task adapted from Long (2020), containing synonym/near-synonym, superordinate-category, and definition items. Children selected the response that best matched the target word. Each correct answer received one point (α = 0.74).

#### 2.3.5 Morphological awareness

Morphological awareness was measured with a compound-word production task adapted from Zhao et al. (2024) and aligned with the productive-compounding task tradition in Chinese literacy research (McBride-Chang et al., 2003). Children heard and saw a short description of a novel referent and wrote the shortest appropriate compound. The task included six practice items, one for each compound structure. For example, children were asked, “What would you call a flower that is hot?” and produced 热花 (“hot flower”). All six practice items are provided in the Supplementary methods. The 24 scored items were organized into six sets, each representing one common Chinese compound structure described in modern Chinese linguistics (Huang and Liao, 2017): adjective-noun, noun-noun, verb-object modifier-noun, coordinate, subject-predicate, and verb-object structures. Standard Mandarin recordings were played to reduce the influence of differences in printed-character recognition. One point was awarded for a semantically appropriate response that conformed to the target compound structure (possible range 0–24; α = 0.75). The task focuses on productive compounding awareness, the facet of Chinese morphological awareness most directly aligned with selecting and combining morphemes into meaningful lexical units.

#### 2.3.6 Phonological awareness

Phonological awareness was assessed using a group-administered paper-and-pencil task adapted from Bai (2017), with 40 items divided equally among rime identification, onset matching, tone identification, and phoneme deletion. In the first three subtests, children selected one of three alternatives that differed from the target in rime, onset, or lexical tone. In the deletion subtest, children removed a specified sound unit from the pronunciation of a target character and wrote a Chinese character corresponding to the remaining pronunciation. Alternative homophonous characters matching that pronunciation were accepted as correct; responses did not have to reproduce the particular character in the answer key. For example, the practice item presented 回 (huí) with the instruction to delete i, and the example answer was 湖 (hú). Standard Mandarin audio accompanied all items. Each correct response received one point (possible range 0–40; α = 0.73). The four subtests sampled sound units relevant to Pinyin and character-pronunciation processing.

#### 2.3.7 Character dictation

The character-dictation task was developed with reference to Shu et al. (2006). It included 50 target characters sampled across frequency levels from the 3,500-character curriculum list (Ministry of Education of the People’s Republic of China, 2022). Each target was presented in a word context through a standard Mandarin recording and was played twice. Children wrote the target character, receiving one point for each correct response (possible range 0–50; α = 0.86).

#### 2.3.8 Syntactic awareness

Syntactic awareness was assessed using a word-order correction task adapted from Yeung (2022) and informed by descriptions of common modern Chinese sentence structures (Huang and Liao, 2017). The task spanned 14 sentence patterns, including adjective-predicate, ba, bei, serial-verb, pivotal, double-object, existential, coordinate, causal, adversative, progressive, conditional, and hypothetical constructions. Children rearranged scrambled words to form a grammatical sentence. The 22 items yielded a maximum of 34 points (α = 0.73). Audio presentation accompanied the printed items.

#### 2.3.9 Non-verbal reasoning and demographic covariates

Non-verbal reasoning was measured with the Chinese revision of Raven’s Standard Progressive Matrices (Zhang and Wang, 1989). The 60 items required selection of the option that completed a visual pattern. One point was awarded for each correct response. Reported split-half and test–retest reliabilities were 0.95 and 0.82, respectively. Grade, sex, and school were included as categorical covariates. Grade 3, girls, and School 1 served as reference categories.

### 2.4 Statistical analysis

Analyses were conducted in R version 4.6.1 on complete data from all 361 eligible participants; there were no missing values in the variables included in the reported models. Throughout this article, sample-standardized scores refer to statistical z transformations of observed raw totals or the specified composites, using the mean and standard deviation of the full study sample [z = (score–sample mean)/sample standard deviation]. These scores were not derived from external age- or grade-normed test scores. Continuous variables were placed on this sample-based scale before regression; dummy-coded demographic variables retained their original coding. HC3 covariance estimation used the sandwich package (version 3.1-2), and robust coefficient tests used the lmtest package (version 0.9-40). Four hierarchical ordinary least-squares models predicted sample-standardized reading-comprehension total scores. Model 1 included grade, sex, school fixed effects, and standardized non-verbal reasoning. Model 2 added the final standardized decoding composite, listening comprehension, and vocabulary. Model 3 added character dictation and syntactic awareness. Model 4 simultaneously added morphological and phonological awareness. Supplementary Table 5 summarizes variable construction and the analytical role of each variable in the primary model sequence.

This approach evaluates associations among performance measures on a common scale while adjusting for grade differences within the regression model. Grade was entered categorically to account for mean differences among Grades 3, 4, and 5. The sensitivity analysis replacing grade with age examined whether the focal comparison was maintained under an alternative representation of developmental background.

Model improvement was evaluated using R^2^, adjusted R^2^, change in R^2^, and the conventional nested-model F-change test. Because unequal residual variance can distort conventional standard errors, coefficient inference used HC3 heteroskedasticity-consistent standard errors (MacKinnon and White, 1985). The joint contribution of morphological and phonological awareness in Model 4 was also evaluated with an HC3 Wald test. Coefficients for continuous predictors are standardized; coefficients for dummy-coded predictors represent differences from the reference category in outcome standard-deviation units. The comparison of morphological and phonological coefficients refers to their conditional standardized associations under the same covariate set. The unique incremental R^2^ of each focal predictor was calculated by removing it from the full model while retaining all other predictors.

The difference between the morphological- and phonological-awareness coefficients was tested directly using the HC3 covariance matrix. A non-parametric case bootstrap with 5,000 resamples was also used for the focal coefficients and their difference (Efron and Tibshirani, 1993). The same resampled cases were used to estimate both coefficients, preserving their dependence. Percentile 95% confidence intervals were computed for each coefficient and for their difference. Multicollinearity was evaluated with variance inflation factors (VIFs). Influence was screened using Cook’s distance with 4/N as a diagnostic threshold; this threshold identified cases for sensitivity analysis rather than automatic deletion.

Three sensitivity analyses were specified. First, age replaced grade. Second, principal-component scores replaced the summed reading-comprehension outcome and the decoding and listening-comprehension composites. Third, the final model was refitted after excluding cases with Cook’s D > 4/N. To examine attenuation and maintain continuity with the original model sequence, a secondary analysis entered morphological and phonological awareness immediately after Model 2 and then added character dictation alone, syntactic awareness alone, or both. All tests were two-sided, with α = 0.05.

OpenAI Codex (GPT-5, OpenAI, San Francisco, CA, United States) was used to assist with data organization, review of locally executed R scripts, and English-language editing. Further details are provided in the Generative AI statement.

## 3 Results

### 3.1 Descriptive statistics and zero-order correlations

Raw-score descriptive statistics by grade are provided in Supplementary Table 1, and zero-order correlations among analytic variables are provided in Supplementary Table 2. Reading comprehension was positively correlated with all focal reading and language variables. The largest correlations were observed for vocabulary knowledge (*r* = 0.644), character dictation (*r* = 0.641), morphological awareness (*r* = 0.624), listening comprehension (*r* = 0.600), and syntactic awareness (*r* = 0.590). Phonological awareness was also moderately correlated with reading comprehension (*r* = 0.537). Morphological and phonological awareness correlated at *r* = 0.403, indicating related but non-redundant skills.

### 3.2 Hierarchical regression models

Model summaries are shown in Table 1. Demographic variables, school, and non-verbal reasoning explained 32.2% of the variance in reading comprehension. Adding decoding, listening comprehension, and vocabulary in Model 2 increased explained variance by 30.7%, ΔF(3, 350) = 96.56, *p* < 0.001. Adding character dictation and syntactic awareness in Model 3 produced a further 5.4% increment, ΔF(2, 348) = 29.54, *p* < 0.001. Morphological and phonological awareness in Model 4 jointly added 2.9% after all covariates, ΔF(2, 346) = 17.65, *p* < 0.001; the HC3 joint Wald test was also significant, χ^2^(2) = 39.50, *p* < 0.001. The final model explained 71.2% of outcome variance (adjusted R^2^ = 0.701).

**TABLE 1:** Hierarchical model fit and incremental variance explained.

Model	Predictors added	R^2^	Adjusted R^2^	Δ R^2^	Δ F	df	*P*
1	Grade, sex, school, non-verbal reasoning	0.322	0.309	0.322	–	–	–
2	Decoding, listening comprehension, vocabulary	0.629	0.619	0.307	96.56	3, 350	<0.001
3	Character dictation, syntactic awareness	0.683	0.672	0.054	29.54	2, 348	<0.001
4	Morphological awareness, phonological awareness	0.712	0.701	0.029	17.65	2, 346	<0.001

R^2^, coefficient of determination; adjusted R^2^, R^2^ adjusted for the number of predictors; ΔR^2^, change in R^2^; ΔF, conventional nested-model F-change statistic.

Table 2 reports coefficients for Models 3 and 4. In Model 3, character dictation (β = 0.248, HC3 SE = 0.048, *p* < 0.001) and syntactic awareness (β = 0.173, HC3 SE = 0.042, *p* < 0.001) both showed unique positive associations. Decoding (β = 0.112, *p* = 0.006), listening comprehension (β = 0.248, *p* < 0.001), and vocabulary (β = 0.260, *p* < 0.001) also remained significant at this stage.

**TABLE 2:** Regression coefficients with HC3 robust standard errors for Models 3 and 4.

Predictor	Model 3 coefficient	HC3 SE	*P*	Model 4 coefficient	HC3 SE	P
Grade 4 (vs. Grade 3)	0.051	0.099	0.610	0.072	0.098	0.466
Grade 5 (vs. Grade 3)	0.135	0.101	0.184	0.060	0.099	0.548
Male (vs. female)	0.038	0.063	0.546	0.052	0.060	0.386
School 2	−0.073	0.108	0.497	−0.115	0.106	0.277
School 3	0.275	0.139	0.048	0.105	0.146	0.475
School 4	0.016	0.105	0.876	−0.039	0.101	0.695
Non-verbal reasoning	0.074	0.039	0.057	0.034	0.038	0.378
Decoding	0.112	0.040	0.006	0.076	0.040	0.057
Listening comprehension	0.248	0.039	<0.001	0.231	0.039	<0.001
Vocabulary knowledge	0.260	0.046	<0.001	0.215	0.045	<0.001
Character dictation	0.248	0.048	<0.001	0.229	0.047	<0.001
Syntactic awareness	0.173	0.042	<0.001	0.108	0.041	0.008
Morphological awareness	–	–	–	0.215	0.036	<0.001
Phonological awareness	–	–	–	0.057	0.044	0.192

β, standardized regression coefficient for continuous predictors; SE, standard error; HC3, heteroskedasticity-consistent covariance estimator type 3. Grade 3, female sex, and School 1 were the reference categories. Intercepts are omitted. Model 3 R^2^ = 0.683; Model 4 R^2^ = 0.712. Continuous scores were standardized within the full study sample, without external age or grade norms.

In Model 4, morphological awareness showed a significant unique association (β = 0.215, HC3 SE = 0.036, *p* < 0.001), with a unique ΔR^2^ of 0.026 and bootstrap 95% CI [0.147, 0.287]. The phonological-awareness coefficient was smaller and non-significant (β = 0.057, HC3 SE = 0.044, *p* = 0.192), with a unique ΔR^2^ of 0.002 and bootstrap 95% CI [−0.027, 0.142]. The direct coefficient contrast favored morphological awareness, βMA−βPA = 0.158, HC3 *p* = 0.009, bootstrap 95% CI [0.043, 0.275]. Character dictation (β = 0.229, *p* < 0.001), listening comprehension (β = 0.231, *p* < 0.001), vocabulary (β = 0.215, *p* < 0.001), and syntactic awareness (β = 0.108, *p* = 0.008) also retained unique positive associations. The final standardized decoding composite was marginal (β = 0.076, HC3 SE = 0.040, *p* = 0.057). Table 3 summarizes the focal coefficients, direct contrasts, and influence-sensitivity results.

**TABLE 3:** Focal coefficients, direct contrasts, and influence sensitivity.

Analysis	*N*	MA β (95% CI)	PA β (95% CI)	β difference	HC3 *p*	95% bootstrap CI for difference
Primary full sample	361	0.215 [0.147, 0.287]	0.057 [−0.027, 0.142]	0.158	0.009	[0.043, 0.275]
Exclude Cook’s D > 4/N	334	0.221 [0.159, 0.283]	0.106 [0.039, 0.170]	0.115	0.023	[0.020, 0.212]

MA, morphological awareness; PA, phonological awareness; CI, percentile 95% confidence interval from 5,000 case-bootstrap resamples. Difference p values use HC3 covariance estimates. All 361 cases were retained in the primary analysis.

### 3.3 Diagnostics and sensitivity analyses

The maximum VIF in the final model was 2.68, providing no indication that severe multicollinearity compromised coefficient estimation. Twenty-seven observations exceeded the Cook’s D diagnostic threshold of 4/N. They were retained in the primary analysis because influence alone is not evidence of error, and their effect was evaluated through sensitivity analysis.

Replacing grade with age yielded a morphological-awareness coefficient of 0.223 (*p* < 0.001), a phonological-awareness coefficient of 0.039 (*p* = 0.347), and a significant coefficient difference (*p* = 0.002). Using principal-component scores for reading comprehension, decoding, and listening comprehension yielded corresponding coefficients of 0.244 (*p* < 0.001) and 0.018 (*p* = 0.686), with a significant difference (*p* < 0.001). After excluding the 27 cases with Cook’s D > 4/N, morphological awareness remained significant (β = 0.221, *p* < 0.001) and phonological awareness also became significant (β = 0.106, *p* = 0.002). The coefficient difference remained significant in this sample, βMA−βPA = 0.115, HC3 *p* = 0.023, bootstrap 95% CI [0.020, 0.212]. Morphological awareness was significant and larger across all sensitivity specifications; inference for the smaller phonological coefficient varied with influential-case handling (Figure 1 and Supplementary Table 4).

**FIGURE 1:**
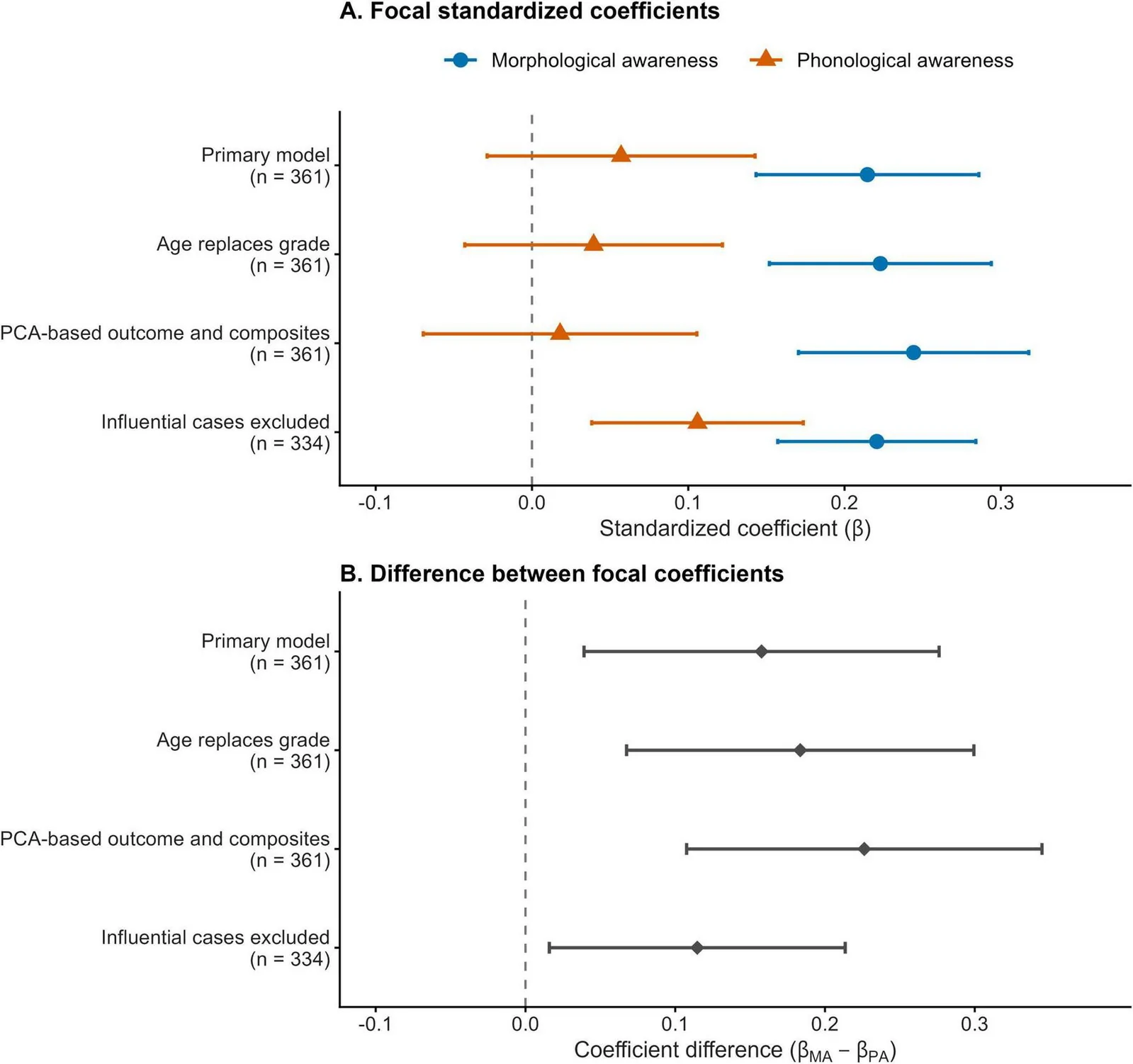
Standardized coefficients for morphological and phonological awareness across the primary and sensitivity models. **(A)** Presents the standardized coefficients for morphological awareness and phonological awareness. **(B)** Presents the corresponding coefficient differences (β MA–β PA). Error bars indicate 95% confidence intervals based on HC3 standard errors. The vertical dashed line indicates zero. PCA, principal component analysis. Standardization refers to the study-sample scale, not external age- or grade-normed scores.

The secondary sequence clarified attenuation of the phonological-awareness coefficient. When morphological and phonological awareness were entered immediately after Model 2, they added 5.0% of explained variance, ΔF(2, 348) = 27.41, *p* < 0.001; morphological awareness was β = 0.241 (*p* < 0.001) and phonological awareness was β = 0.134 (*p* = 0.002). Adding character dictation alone reduced the phonological coefficient to 0.068 (*p* = 0.121), while morphological awareness remained 0.238 (*p* < 0.001). Adding syntactic awareness alone yielded β = 0.116 for phonological awareness (*p* = 0.007) and β = 0.212 for morphological awareness (*p* < 0.001). The principal reduction in the phonological coefficient was associated with character dictation (Supplementary Table 4).

## 4 Discussion

This study compared the unique associations of morphological and phonological awareness with reading comprehension in Chinese children in Grades 3–5. Three findings stand out. First, after demographic, cognitive, reading, character-level, and language covariates were entered, the two metalinguistic skills jointly explained an additional 2.9% of variance. Second, morphological awareness retained a stable unique association across all specifications. Third, phonological awareness showed a smaller unique association whose significance varied with influential-case handling, while the direct morphological-phonological coefficient difference remained significant in both the full sample and the sample after influential-case exclusion. The pattern supports a larger and more robust conditional association for morphological awareness within Grades 3–5.

### 4.1 Morphological awareness as a robust predictor

The morphological-awareness result is consistent with the linguistic structure of Chinese and with prior longitudinal and meta-analytic evidence (Cheng et al., 2016; Dong et al., 2020; Ke, 2025; Wang et al., 2023; Xie et al., 2019). The compound-production task required children to select morpheme meanings and combine them according to productive word-formation patterns. These operations connect word identification with meaning construction: constituents organize multi-character words, their relations guide interpretation of compounds, and context helps select the relevant meaning. Kim et al. (2020) identified direct and indirect compounding associations through vocabulary, word reading, and listening comprehension in Chinese second graders. The present findings extend this line of inquiry to Grades 3–5 and show that productive compounding retains a stable unique association after character dictation and syntactic awareness are also represented.

The coefficient remained significant after vocabulary knowledge was controlled. Vocabulary breadth indexes the amount of lexical knowledge already available, whereas productive compounding requires children to organize constituent meanings into a new, structurally appropriate lexical unit. This distinction explains why a measure of existing word knowledge may leave additional comprehension-related variation associated with flexible meaning construction. In the final model, vocabulary and morphological awareness had comparable standardized coefficients (both approximately 0.215), indicating distinct conditional associations with comprehension after decoding, listening comprehension, character dictation, syntax, non-verbal reasoning, and school differences were considered. The Lexical Constituency Model provides a character-level basis for this interpretation: access to meaning is coordinated with orthographic and phonological constituents (Perfetti et al., 2005). Productive compounding builds on access to those meanings by organizing relations among morphemes at the word level.

The finding complements Wang et al. (2023), who reported that compounding awareness uniquely predicted reading comprehension among Chinese children and that its role varied with grade and reading level. It also aligns with evidence from Grades 3–5 that morphological awareness remains a consistent indicator of reading comprehension across upper-elementary grade groups (Cheng and Zhang, 2023). Longitudinal evidence of reciprocal, time-sensitive relations between morphological awareness and Chinese reading skills (Wang et al., 2025) and meta-analytic evidence identifying task-related variation in morphological associations (Dong et al., 2020; Ke, 2025; Ruan et al., 2018) further situate the present productive-compounding result. The current analysis adds a common, fully adjusted comparison of morphological and phonological awareness. Its contribution is to locate productive meaning construction within a reading system already represented by measures of decoding, language comprehension, character-level literacy, and sentence structure.

The cross-sectional coefficient establishes a concurrent association and does not determine causal direction. Reading comprehension may also foster morphological awareness by exposing children to varied compound structures and contexts for inferring morpheme meanings. Longitudinal studies have reported reciprocal relations (Cheng et al., 2016; Wang et al., 2025; Xie et al., 2019). Longitudinal and intervention designs are needed to distinguish these directions.

### 4.2 A smaller and shared phonological-awareness contribution

Phonological awareness showed a meaningful association with Chinese reading comprehension. Its zero-order correlation with reading comprehension was 0.537, and in the secondary original-order model it retained a unique coefficient after demographic, decoding, listening-comprehension, and vocabulary controls (β = 0.134, *p* = 0.002). Rime, onset, tone, and deletion awareness can support Pinyin knowledge, pronunciation retrieval, and the establishment of character representations. The fully adjusted coefficient was smaller after character dictation and syntactic awareness were represented.

The secondary-sequence analysis identifies character-level literacy as an important context for understanding the phonological association. Syntactic awareness alone did not eliminate that association, whereas character dictation reduced it by roughly half. Dictation begins with an auditory word and requires the child to identify the target pronunciation, access its meaning in context, select the appropriate character among homophones, and retrieve a precise orthographic form. It therefore samples the coordination of phonological, orthographic, and semantic information described by the Lexical Constituency Model (Perfetti et al., 2005). The observed attenuation is consistent with phonological awareness and dictation sharing aspects of this character-processing foundation. Written responses in the deletion subtest provide an additional source of overlap. The contrast between dictation and syntax shows why the particular literacy abilities represented in a model matter for interpreting the remaining phonological association.

Developmental stage may also matter. The sample consisted of children in Grades 3–5. Research with Mandarin-speaking school-age children indicates that syllable awareness reaches high levels relatively early, while onset-rime awareness and Pinyin knowledge continue to develop (Lin et al., 2020). By the middle primary years, character recognition and Pinyin-supported phonological decoding are more practiced than in beginning readers. Variation in comprehension may consequently depend more strongly on oral-language and semantic integration. Phonological awareness can still operate through established decoding and lexical representations even when its fully adjusted coefficient is small.

Across the sensitivity analyses, morphological awareness remained significant and larger than phonological awareness. After cases exceeding Cook’s D > 4/N were excluded, phonological awareness also became significant, while the morphological-phonological coefficient difference remained significant (β difference = 0.115, HC3 *p* = 0.023; bootstrap 95% CI [0.020, 0.212]). The comparative result therefore does not depend on treating phonological awareness as null. It reflects the larger and more stable unique association of morphological awareness across the reported specifications.

### 4.3 Reading and language foundations

The broader regression pattern connects the study to the SVR account of Chinese reading comprehension. Decoding, listening comprehension, and vocabulary together added 30.7% of explained variance beyond demographic, school, and non-verbal-reasoning variables. This result establishes the measured reading and language foundation against which the focal skills were evaluated (Hoover and Gough, 1990; Peng et al., 2021). Listening comprehension and vocabulary remained strong unique predictors in the final model, linking comprehension to the construction of meaning from language and access to lexical knowledge.

Listening comprehension and character dictation had the largest final-model coefficients, followed closely by vocabulary and morphological awareness. Character dictation is a demanding integrated literacy task and may indicate the precision of children’s lexical representations. Decoding remained significant in Model 3 but became marginal after morphological and phonological awareness were added in Model 4. Syntactic awareness made a smaller but significant contribution, supporting evidence that Chinese word order and morphosyntactic knowledge assist sentence integration (Chik et al., 2012; Yeung et al., 2013).

The study refines a componential account of Chinese reading by identifying different patterns of association for productive compounding and phonological analysis within the same reading and language framework. Peng et al. (2021) found that the broad metalinguistic contribution was represented by decoding and language comprehension, while Kim et al. (2020) identified additional compounding associations. Our results show that productive compounding retains a stable unique association even with character-level literacy and sentence structure represented. The phonological association is more closely tied to the character-level abilities included in the comparison. These findings give concrete content to the question of which skills are captured by broad reading and language measures. They also fit DIER’s emphasis on the developmental and assessment conditions under which component relations are observed (Kim, 2020). Here, the observed models specify that organization through incremental and shared associations; the proposed pathways provide a framework for longitudinal tests of how those relations develop.

### 4.4 Educational implications

These findings suggest differentiated instructional emphases for children in Grades 3–5. The larger and more stable unique association of compounding awareness with reading comprehension provides a rationale for incorporating morpheme meanings and compound-word formation into vocabulary and comprehension instruction. Teachers could guide students to analyze the meanings of constituent morphemes, compare words that share a morpheme, generate and explain novel compounds, and use compound structure to infer the meanings of unfamiliar words in context.

Phonological awareness showed a smaller association that was largely shared with decoding and character dictation, indicating that its educational relevance at this stage is closely tied to character-level literacy. Phonological analysis can be incorporated into character recognition, pronunciation, and writing activities that connect syllable, onset-rime, and tone information with orthographic form and word meaning. Assessment can likewise consider phonological awareness together with decoding and dictation when identifying the component skills associated with reading-comprehension difficulties and planning instructional support.

### 4.5 Limitations and future directions

(1) The cross-sectional design cannot establish temporal or causal direction. Although HC3 standard errors, bootstrapping, diagnostic checks, and sensitivity models improved inferential robustness, the result for phonological awareness remained sensitive to influential-case handling. Replication should therefore focus on effect sizes and confidence intervals rather than dichotomous significance alone. Longitudinal models should examine whether morphological and phonological awareness predict subsequent change in comprehension after prior comprehension is controlled. Intervention studies could compare morphology-focused, phonology-focused, and integrated instruction.

(2) Participants came from four schools in two cities in one province. School fixed effects controlled for mean differences across the four schools, but the small number of schools precluded reliable cluster-robust or multilevel estimation; residual dependence within schools therefore cannot be ruled out. Generalization to other regions, dialect backgrounds, curricula, or orthographic traditions requires replication. Future research should sample a larger number of schools to permit explicit estimation of classroom- and school-level variation.

(3) Each focal construct was represented by one observed total score: compound production for morphological awareness and a combination of rime, onset, tone, and deletion tasks for phonological awareness. Differential measurement reliability or task demands can influence coefficient magnitude; grade-specific internal-consistency estimates are reported in Supplementary Table 3. The deletion subtask required a written-character response, so performance may partly reflect orthographic retrieval alongside phonological manipulation. Character dictation is also broad: its shared variance with phonological awareness may reflect pronunciation processing, character-specific orthography, semantic selection among homophones, or general literacy proficiency. These overlapping demands complicate interpretation of the attenuation of phonological awareness after character dictation was added. Future studies should use multiple indicators, include phonological tasks without written responses, and distinguish orthographic awareness, character writing fluency, and lexical-quality components to compare specific facets and clarify the attenuation mechanism.

(4) The use of sample-standardized scores also shapes the developmental interpretation of the findings. Our sample-standardized scores locate each child relative to the full study sample, with grade differences adjusted in the regression. Age- or grade-normed scores instead locate performance relative to an appropriate developmental reference group (Lenhard et al., 2019). Differences in score dispersion across groups and in the norming transformation could change the relative scaling of skills, their coefficients, and their incremental associations with comprehension. The age-covariate sensitivity analysis maintained the main comparison. Future studies using suitable age- or grade-normed measures can extend this evidence by testing whether the stronger morphological association also characterizes children’s relative standing among same-age or same-grade peers.

## 5 Conclusion

Chinese reading comprehension in Grades 3–5 is associated with metalinguistic skills embedded in a broader system of word identification, language understanding, and character knowledge. Productive compounding retained a stable unique association within this system, indicating that the ability to organize morphemes into meaningful words provides comprehension-related information beyond the reading and language measures considered here. Phonological awareness showed a smaller association that was closely connected to character-level literacy and varied with influential-case handling. Together, these findings specify how meaning construction and sound analysis are situated within the foundations described by the Simple View of Reading. They give particular prominence to flexible compound-meaning construction while locating phonological awareness within the coordinated processing of character forms, pronunciations, and meanings. For instruction and assessment in these grades, the findings support attention to children’s use of morpheme meanings and compound structure alongside an integrated understanding of their character recognition, pronunciation, and writing skills.

## Data Availability

The datasets presented in this article are not readily available because they concern minors and are subject to ethical and privacy restrictions. Requests for de-identified data will be considered subject to applicable ethics approval, privacy safeguards, and a data use agreement. Requests to access the datasets should be directed to Xueru Zhang, 52204109004@stu.ecnu.edu.cn.

## References

[B1] Bai L.-R. (2017). *A New Approach to Detecting Reading Disabilities and Identifying Subtypes.* Tianjin: Nankai University Press.

[B2] Center for Language Education and Cooperation (2026). *Chinese Test Service Website.* Beijing: Center for Language Education and Cooperation.

[B3] Cheng X. Zhang H. S. (2023). Contributions of metalinguistic awareness and lexical inference to reading comprehension in upper-elementary Chinese students. *J. Res. Read.* 46, 86–103. 10.1111/1467-9817.12415

[B4] Cheng Y. Zhang J. Wu X. Liu H. Li H. (2016). Cross-lagged relationships between morphological awareness and reading comprehension among Chinese children. *Front. Psychol.* 7:1379. 10.3389/fpsyg.2016.01379 27708594 PMC5030266

[B5] Chik P. P.-M. Ho C. S.-H. Yeung P.-S. Chan D. W.-O. Chung K. K.-H. Luan H. et al. (2012). Syntactic skills in sentence reading comprehension among Chinese elementary school children. *Read. Writ.* 25, 679–699. 10.1007/s11145-010-9293-4

[B6] Dong Y. Peng S.-N. Sun Y.-K. Wu S. X.-Y. Wang W.-S. (2020). Reading comprehension and metalinguistic knowledge in Chinese readers: A meta-analysis. *Front. Psychol.* 10:3037. 10.3389/fpsyg.2019.03037 32116868 PMC7013083

[B7] Efron B. Tibshirani R. J. (1993). *An Introduction to the Bootstrap.* New York, NY: Chapman & Hall/CRC.

[B8] Geng Y.-J. (2014). *Measurement and Intervention of Morphological Awareness in Chinese Children with Developmental Reading Difficulties.* Master’s thesis, Xiamen: Xiamen University.

[B9] Guan C. Q. Liu Y. Chan D. H. L. Ye F. Perfetti C. A. (2011). Writing strengthens orthography and alphabetic-coding strengthens phonology in learning to read Chinese. *J. Educ. Psychol.* 103, 509–522. 10.1037/a0023730

[B10] Han B. Koh P. W. Zhang S. Joshi R. M. Li H. (2022). The relative contributions of facets of morphological awareness to vocabulary development in Chinese: A longitudinal study in grades one to three. *Contemp. Educ. Psychol.* 69:102063. 10.1016/j.cedpsych.2022.102063

[B11] Ho C. S.-H. Chan D. W.-O. Lee S.-H. Tsang S.-M. Luan V. H. (2004). Cognitive profiling and preliminary subtyping in Chinese developmental dyslexia. *Cognition* 91, 43–75. 10.1016/S0010-0277(03)00163-X 14711491

[B12] Ho C. S.-H. Zheng M. McBride C. Hsu L. S. J. Waye M. M. Y. Kwok J. C. Y. (2017). Examining an extended simple view of reading in Chinese: The role of naming efficiency for reading comprehension. *Contemp. Educ. Psychol.* 51, 293–302. 10.1016/j.cedpsych.2017.08.009

[B13] Hoover W. A. Gough P. B. (1990). The simple view of reading. *Read. Writ.* 2, 127–160. 10.1007/BF00401799

[B14] Huang B.-R. Liao X.-D. (2017). *Modern Chinese*, 6th Edn. Beijing: Higher Education Press.

[B15] Ke S. (2025). Chinese morphological awareness assessment and its relation to reading acquisition: A cross-cultural meta-analysis. *Humanit. Soc. Sci. Commun.* 12:235. 10.1057/s41599-025-04531-6

[B16] Kim Y. S. G. (2020). Toward integrative reading science: The direct and indirect effects model of reading. *J. Learn. Disabil.* 53, 469–491. 10.1177/0022219420908239 32125226

[B17] Kim Y. S. G. (2023). Executive functions and morphological awareness explain the shared variance between word reading and listening comprehension. *Sci. Stud. Read.* 27, 451–474. 10.1080/10888438.2023.2195112 37600965 PMC10438421

[B18] Kim Y. S. G. Guo Q. Liu Y. Peng Y. Yang L. (2020). Multiple pathways by which compounding morphological awareness is related to reading comprehension: Evidence from Chinese second graders. *Read. Res. Q.* 55, 193–212. 10.1002/rrq.262

[B19] Ku Y.-M. Anderson R. C. (2003). Development of morphological awareness in Chinese and English. *Read. Writ.* 16, 399–422. 10.1023/A:1024227231216

[B20] Lenhard A. Lenhard W. Gary S. (2019). Continuous norming of psychometric tests: A simulation study of parametric and semi-parametric approaches. *PLoS One* 14:e0222279. 10.1371/journal.pone.0222279 31527877 PMC6748442

[B21] Lin Y. Lin Y.-J. Wang F. Wu X. Kong J. (2020). The development of phonological awareness and Pinyin knowledge in Mandarin-speaking school-aged children. *Int. J. Speech Lang. Pathol.* 22, 660–668. 10.1080/17549507.2020.1819417 33059483

[B22] Liu P. D. McBride-Chang C. (2010). What is morphological awareness? Tapping lexical compounding awareness in Chinese third graders. *J. Educ. Psychol.* 102, 62–73. 10.1037/a0016933

[B23] Liu P. D. McBride-Chang C. Wong T. T.-Y. Shu H. Wong A. M.-Y. (2013). Morphological awareness in Chinese: Unique associations of homophone awareness and lexical compounding to word reading and vocabulary knowledge in Chinese children. *Appl. Psycholinguist.* 34, 755–775. 10.1017/S014271641200001X

[B24] Long Y.-L. (2020). *The Role of Morphological Awareness in the Reading Ability of Primary School Students with Chinese Reading Disabilities.* Master’s thesis, Chongqing: Southwest University.

[B25] MacKinnon J. G. White H. (1985). Some heteroskedasticity-consistent covariance matrix estimators with improved finite sample properties. *J. Econom.* 29, 305–325. 10.1016/0304-4076(85)90158-7

[B26] McBride-Chang C. Shu H. Zhou A. Wat C. P. Wagner R. K. (2003). Morphological awareness uniquely predicts young children’s Chinese character recognition. *J. Educ. Psychol.* 95, 743–751. 10.1037/0022-0663.95.4.743

[B27] Meng X.-Z. Li C.-Z.-J. (2024). Development, validity, and reliability of an assessment for Chinese developmental dyslexia [in Chinese]. *Chin. Ment. Health J.* 38, 706–712. 10.3969/j.issn.1000-6729.2024.08.011

[B28] Ministry of Education of the People’s Republic of China (2022). *Chinese Language Curriculum Standards for Compulsory Education (2022 edition).* Beijing: Beijing Normal University Publishing Group.

[B29] Modern Chinese Common Word List Research Group (2008). *Modern Chinese Common Word List: Draft.* Beijing: The Commercial Press.

[B30] Peng P. Lee K. Luo J. Li S. Joshi R. M. Tao S. (2021). Simple view of reading in Chinese: A one-stage meta-analytic structural equation modeling. *Rev. Educ. Res.* 91, 3–33. 10.3102/0034654320964198

[B31] Perfetti C. A. Liu Y. Tan L. H. (2005). The lexical constituency model: Some implications of research on Chinese for general theories of reading. *Psychol. Rev.* 112, 43–59. 10.1037/0033-295X.112.1.43 15631587

[B32] Ruan Y. Georgiou G. K. Song S. Li Y. Shu H. (2018). Does writing system influence the associations between phonological awareness, morphological awareness, and reading? A meta-analysis. *J. Educ. Psychol.* 110, 180–202. 10.1037/edu0000216

[B33] Shu H. McBride-Chang C. Wu S. Liu H. (2006). Understanding Chinese developmental dyslexia: Morphological awareness as a core cognitive construct. *J. Educ. Psychol.* 98, 122–133. 10.1037/0022-0663.98.1.122

[B34] Wang Z. Ding W. Xie R. Wu X. Wenren S. Xia Y. (2025). Exploring the dynamic interplays of morphological awareness and reading skills in Chinese children. *Child Dev.* 96, 1017–1033. 10.1111/cdev.14214 39825579

[B35] Wang Z. Xie R. Wu X. Xia Y. Yu Y. Nguyen T. P. et al. (2023). Unpacking the relation between morphological awareness and reading comprehension in Chinese children. *Br. J. Educ. Psychol.* 93, 773–789. 10.1111/bjep.12591 36774949

[B36] Wen H.-B. (2005). *Development of a Scale for Assessing Primary School Students’ Chinese Reading Ability.* Master’s thesis, Guangzhou: South China Normal University.

[B37] Xie R. Zhang J. Wu X. Nguyen T. P. (2019). The relationship between morphological awareness and reading comprehension among Chinese children. *Front. Psychol.* 10:54. 10.3389/fpsyg.2019.00054 30774607 PMC6367908

[B38] Yeung P.-S. (2022). A model of Chinese reading comprehension: The role of cognition and motivation. *Learn. Individ. Differ.* 99:102202. 10.1016/j.lindif.2022.102202

[B39] Yeung P.-S. Ho C. S.-H. Chan D. W.-O. Chung K. K.-H. Wong Y.-K. (2013). A model of reading comprehension in Chinese elementary school children. *Learn. Individ. Differ.* 25, 55–66. 10.1016/j.lindif.2013.03.004

[B40] Zhang H.-C. Wang X.-P. (1989). Standardization research on Raven’s standard progressive matrices in China. *Acta Psychol. Sin.* 21, 113–121.

[B41] Zhao Y. Wu X.-C. Chen H.-J. Sun P. Wang H.-L. (2024). Effects of morphological awareness and rapid naming on Chinese children’s reading abilities: Mediation across grade levels. *Acta Psychol. Sin.* 56, 70–82. 10.3724/SP.J.1041.2024.00070

