# Supplementary material for: Morphological awareness shows a more robust unique association with reading comprehension than phonological awareness in Chinese children in Grades 3–5
Source: Front Psychol. 2026 Sep 21;17:1946945. doi: 10.3389/fpsyg.2026.1946945 (PMC13635111; doi:10.3389/fpsyg.2026.1946945)
Supplement: Supplementary file 1 [file Table_1.DOCX]

Supplementary Material

# Supplementary Methods

Sample-standardized scores are z transformations based on the mean and standard deviation of the full study sample. Observed raw totals or the specified composites were used; no external age- or grade-norm conversion was applied. All analyses used the same coding and standardization rules as the primary models. The three timed decoding scores were standardized separately, averaged with equal weights, and the resulting composite was standardized again before regression. In the age sensitivity model, sample-standardized age replaced the two grade indicators. Principal-component sensitivity scores were constructed separately for reading comprehension, decoding, and listening comprehension, with component orientation set so that higher values represented stronger performance. The influential-case analysis removed observations with Cook's D > 4/N only for sensitivity assessment; all observations were retained in the primary analysis. The secondary sequence entered morphological and phonological awareness immediately after Model 2 and then added character dictation, syntactic awareness, or both to describe coefficient attenuation.

## Compound-production practice items

The task included one practice item for each of the six compound structures: (1) adjective-noun, 热热的花，叫什么？ ('What would you call a flower that is hot?'), 热花 ('hot + flower'); (2) noun-noun, 用猴子拉的车，叫什么？ ('What would you call a cart pulled by a monkey?'), 猴车 ('monkey + cart'); (3) verb-object modifier-noun, 煮鲜花的锅，叫什么？ ('What would you call a pot used to cook flowers?'), 煮花锅 ('cook-flower + pot'); (4) coordinate, 鱼和花放在一起，叫什么？ ('What would you call a fish and a flower placed together?'), 鱼花 ('fish + flower'); (5) subject-predicate, 小马在跳舞，叫什么？ ('What would you call a horse dancing?'), 马舞 ('horse + dance'); and (6) verb-object, 把瓶子锁起来，叫什么？ ('What would you call locking a bottle?'), 锁瓶 ('lock + bottle').

Supplementary Table S1. Raw-score descriptive statistics for the full sample and by grade.

| **Variable** | **Full sample (N = 361)** | **Grade 3 (n = 108)** | **Grade 4 (n = 168)** | **Grade 5 (n = 85)** |
| --- | --- | --- | --- | --- |
| Age (years) | 9.68 (0.80) | 8.70 (0.33) | 9.80 (0.28) | 10.66 (0.43) |
| Reading comprehension total | 27.00 (7.76) | 22.32 (7.58) | 28.14 (6.64) | 30.69 (7.27) |
| Nonverbal reasoning (RSPM) | 35.48 (9.35) | 33.35 (9.36) | 36.13 (9.37) | 36.92 (8.91) |
| Timed word reading | 78.22 (23.01) | 67.45 (18.96) | 81.02 (22.97) | 86.36 (23.03) |
| Timed character reading | 91.64 (24.13) | 85.21 (23.69) | 91.09 (23.20) | 100.89 (23.89) |
| Timed Pinyin reading | 31.27 (15.85) | 27.41 (11.87) | 29.95 (15.12) | 38.79 (19.03) |
| Listening comprehension total | 13.97 (3.29) | 12.48 (3.21) | 14.34 (3.08) | 15.12 (3.14) |
| Vocabulary knowledge | 24.37 (7.23) | 20.33 (6.41) | 25.64 (7.23) | 26.98 (6.07) |
| Morphological awareness | 9.93 (4.77) | 7.64 (4.57) | 10.23 (4.40) | 12.22 (4.46) |
| Phonological awareness | 28.56 (9.90) | 27.39 (10.01) | 28.94 (9.87) | 29.27 (9.79) |
| Character dictation | 30.45 (10.86) | 22.70 (8.07) | 32.70 (10.16) | 35.84 (10.03) |
| Syntactic awareness | 13.46 (5.51) | 12.00 (4.50) | 13.76 (5.71) | 14.71 (5.92) |

Note. Values are M (SD). All performance variables are raw totals or correct-item counts. The three raw timed-reading scores are displayed instead of the sample-standardized decoding composite so that the descriptive scale remains interpretable.

Supplementary Table S2. Zero-order correlations among analytic continuous variables (N = 361).

| **Variable** | **1** | **2** | **3** | **4** | **5** | **6** | **7** | **8** | **9** |
| --- | --- | --- | --- | --- | --- | --- | --- | --- | --- |
| 1. Reading comprehension | – | .431 | .521 | .600 | .644 | .624 | .537 | .641 | .590 |
| 2. Nonverbal reasoning | .431 | – | .189 | .304 | .455 | .396 | .438 | .232 | .524 |
| 3. Decoding composite | .521 | .189 | – | .333 | .401 | .427 | .270 | .540 | .433 |
| 4. Listening comprehension | .600 | .304 | .333 | – | .439 | .357 | .409 | .432 | .359 |
| 5. Vocabulary knowledge | .644 | .455 | .401 | .439 | – | .474 | .553 | .417 | .563 |
| 6. Morphological awareness | .624 | .396 | .427 | .357 | .474 | – | .403 | .416 | .519 |
| 7. Phonological awareness | .537 | .438 | .270 | .409 | .553 | .403 | – | .424 | .503 |
| 8. Character dictation | .641 | .232 | .540 | .432 | .417 | .416 | .424 | – | .416 |
| 9. Syntactic awareness | .590 | .524 | .433 | .359 | .563 | .519 | .503 | .416 | – |

Note. The decoding composite is the final sample-standardized equal-weight mean of separately sample-standardized Pinyin-, character-, and word-reading scores. All displayed correlations are significant at p < .001.

Supplementary Table S3. Internal-consistency reliability by grade and in the full sample.

| **Measure** | **Grade 3 (n = 108)** | **Grade 4 (n = 168)** | **Grade 5 (n = 85)** | **Full sample (N = 361)** |
| --- | --- | --- | --- | --- |
| Text comprehension | .815 | .794 | .809 | .830 |
| Sentence comprehension | .773 | .727 | .629 | .740 |
| Paragraph comprehension | .765 | .648 | .698 | .740 |
| Vocabulary knowledge | .669 | .740 | .631 | .740 |
| Morphological awareness | .728 | .707 | .715 | .750 |
| Phonological awareness | .736 | .729 | .724 | .730 |
| Character dictation | .747 | .840 | .836 | .860 |
| Listening comprehension: sentence | .767 | .775 | .756 | .790 |
| Listening comprehension: dialogue | .750 | .643 | .674 | .710 |
| Syntactic awareness | .615 | .718 | .766 | .730 |

Note. Values are Cronbach's α.

Supplementary Table S4. Sensitivity analyses and secondary-sequence attenuation of the focal coefficients.

**A. Prespecified sensitivity analyses**

| **Analysis** | **N** | **R²** | **Morphological β (p)** | **Phonological β (p)** | **β difference (HC3 p)** |
| --- | --- | --- | --- | --- | --- |
| Age replaces grade | 361 | .712 | .223 (< .001) | .039 (.347) | .183 (.002) |
| PCA-based outcome and composites | 361 | .696 | .244 (< .001) | .018 (.686) | .226 (< .001) |
| Exclude Cook's D > 4/N | 334 | .791 | .221 (< .001) | .106 (.002) | .115 (.023) |

**B. Secondary original-order attenuation analyses**

| **Analysis** | **R²** | **Morphological β (p)** | **Phonological β (p)** | **β difference (HC3 p)** |
| --- | --- | --- | --- | --- |
| Original Model 3 | .680 | .241 (< .001) | .134 (.002) | .107 (.086) |
| Original Model 3 + character dictation | .707 | .238 (< .001) | .068 (.121) | .170 (.005) |
| Original Model 3 + syntactic awareness | .688 | .212 (< .001) | .116 (.007) | .096 (.118) |
| Full model | .712 | .215 (< .001) | .057 (.192) | .158 (.009) |

Note. β = standardized regression coefficient; HC3 = heteroskedasticity-consistent covariance estimator type 3; PCA = principal component analysis. The primary analysis uses the reordered sequence in which character dictation and syntactic awareness enter before the focal predictors.

Supplementary Table S5. Variable construction and analytical role.

| **Variable** | **Construction or scoring** | **Role in primary analysis** |
| --- | --- | --- |
| Reading comprehension | Sum of 42 sentence-, paragraph-, and text-level items; sample-standardized | Outcome |
| Nonverbal reasoning | Raven's Standard Progressive Matrices total; sample-standardized | Model 1 covariate |
| Decoding | Pinyin, character, and word scores separately sample-standardized; equal-weight mean sample-standardized again | Model 2 covariate |
| Listening comprehension | Sum of sentence and dialogue tasks; sample-standardized | Model 2 covariate |
| Vocabulary knowledge | 40-item vocabulary breadth total; sample-standardized | Model 2 covariate |
| Character dictation | 50-item dictated-character total; sample-standardized | Model 3 covariate |
| Syntactic awareness | 22-item word-order correction score; sample-standardized | Model 3 covariate |
| Morphological awareness | 24-item compound-word production total; sample-standardized | Model 4 focal predictor |
| Phonological awareness | 40-item rime, onset, tone, and deletion total; sample-standardized | Model 4 focal predictor |
| Grade | Grade 3 reference; indicators for Grades 4 and 5 | Model 1 covariate |
| Sex | Female reference; male indicator | Model 1 covariate |
| School | School 1 reference; indicators for Schools 2–4 | Model 1 fixed effects |

Note. Sample-standardized denotes a z score based on the full study sample, not an external age- or grade-normed standard score. The decoding subtest scores were standardized separately, averaged with equal weights, and the composite was standardized again.
